# Supplementary material for: An open platform for visual stimulation of insects
Source: PLoS One. 2024 Apr 18;19(4):e0301999. doi: 10.1371/journal.pone.0301999 (PMC11025907; doi:10.1371/journal.pone.0301999)
Supplement: Attachment — Submitted filename: Response to Reviewers.pdf [file pone.0301999.s001.pdf]

Response to Reviewers
# of manuscript PONE-D-24-00888

We are grateful to our referees for the time and the effort they invested in the assessment of our work. The revised manuscript is improved considerably thanks to their advice.

Below please find a point-by-point response to their comments.

Reviewer #1:

The manuscript presented by Prech et al. describes a novel, versatile visual stimulation setup for insects and demonstrates its feasibility for studying response properties of visual neurons as well as visually guided behaviors. The authors provide a detailed description of the screen properties, highlighting advantages compared to other established visual stimulation methods and geometries in the field. The relatively small dimensions, large field of view and reduced computational cost due to the precalculated screen geometry make this setup a great option for many labs studying insect vision.
The manuscript is technically sound and written in an intelligible fashion. The data supports the authors conclusions with the statistical analysis being done in accordance with current scientific standards.

I only have a few minor comments:

1. Is the contrast the system can deliver comparable to other visual stimulation devices like LED matrices or liquid cristal devices?
Maybe the authors could add a few thoughts on this to the discussion.

> We added a critical discussion of the contrast of the system in lines 399-410 of the revised manuscript, including a comparison with other commonly used visual stimulation devices.

2. While reading the manuscript it didn't become clear to me how well a fly is shielded from direct exposure to the projector beams. Eg would a fly placed in the fly holder in fig.1 be exposed to direct projector light from the back as well (creating large differences in the perceived intensity coming from the screen vs the projector)? Or would this effect be minimized by adding dark areas to the projection image according to a fly's position within the setup? I'd find it helpful if the authors could add a bit more clarification on this.

Screen areas that would expose the fly to illumination from the projector were masked so that no direct light hits the fly. We added this information to the revised version of the manuscript (lines 182-185).

L16:...repeated presentations of the same stimulus tend to elicit different neural responses. Please add reference

> We added the relevant references.

L452: dot is part of hyperlink and results in broken link.

> We corrected the link.

Fig1a-c: what does the purple hexagon indicate? What do the arrows on the gray hexagon indicate?

> We edited Fig 1 for clarity. It is now free from distracting arrows and the caption contains an explanation of the reference hexagon.

Reviewer #2:

I congratulate the authors on the thorough and meticulous work, clear presentation and an accessible manuscript that was a pleasure to read. The article will be an excellent guide. I have no major remarks and the manuscript can be accepted as is. However, to broaden the impact and guide the future builders, it may be useful to consider the following:

1. Is the intensity of projection system linear, easy to control, suitable for presenting arbitrary contrasts? What are the maximum and minimum contrast ratios? I know this largely depends on the projector used, but it is useful to remind people that it may be an issue.

> In lines 399-410 of the revised manuscript, we added a paragraph on contrast ratios and their dependency on the type of projector used. The new section also contains a comparison with other commonly used display devices.

2. Insect folks will be using UV capable projectors. In this case, the paint and varnish used here may not be appropriate. It would be good, but not strictly necessary, to know, if this particular combination is UV-reflective, UV-absorptive or perhaps even fluorescent.

> This is an important point that we tried to heed in the revised version of the manuscript: We state explicitly the spectral conditions under which the coating was evaluated, and performed an additional test under UV conditions. The results are described in lines 503-507 of the revised manuscript.

3. A large piece of plastic such as the bowl in a dry lab may become charged with static electricity and even damage the ephys headstage. It could be wise to ground it just in case.

> We neither detected any significant electrostatic charges when using the Super Bowl, nor did the headstage of our patch clamp amplifier show any damage throughout extensive testing. In our hands, a metal base plate was sufficient. However, if required, a wire mesh on the outside of the bowl would both ground the screen and provide shielding from ambient electric noise. We added a passage to lines 517-519 the manuscript to clarify this point.

4. The angular resolution of Drosophila eyes is about isotropic across the visual field. Many other insects have regions named as acute zones with locally higher resolution. A projection system should match the highest angular resolution achieved in any compound eye. A reader might benefit from being aware of this.

We added a passage to the Methods section (lines 532-539) in which we describe both the resolution of the virtual texture and the optical resolution of the projector system in more detail. The new section ends with a statement concerning the suitability of our system to stimulate insect species with acute zones of higher spatial resolution.

Other comments:

L16 photoreceptors are very reliable and have extremely reproducible responses, the uncertainty probably accumulates downstream the visual pathway. [Please ignore this remark if you wish as the text flow is very nice here]

Thank you for the thoughtful comment. In this case, we prefer to follow the suggestion in the addendum and stick to the narrative. We added references to strengthen the statement that—downstream of photoreceptors—visual responses are inherently variable. Precise stimulation is a prerequisite to identify sources of uncertainty, even if the front end encodes visual stimuli with utmost fidelity.

L43 I thought that the custom curved LED displays cover a substantial part of the visual field, IDK...

Especially under microscopic conditions, commonly used LED displays usually stimulate only a small strip-shaped section of the visual field. Isaacson *et al.* (*BioRxiv* 2022) tried to address this limitation, for example, by using a motorized rotational stage to allow for the sequential stimulation of different sections. Below, in Fig 4C of Isaacson *et al.*, the LED screen (green) is projected onto an equirectangular grid at three different positions. This can be directly compared to the bowl-shaped screen in Fig 6C of our manuscript.

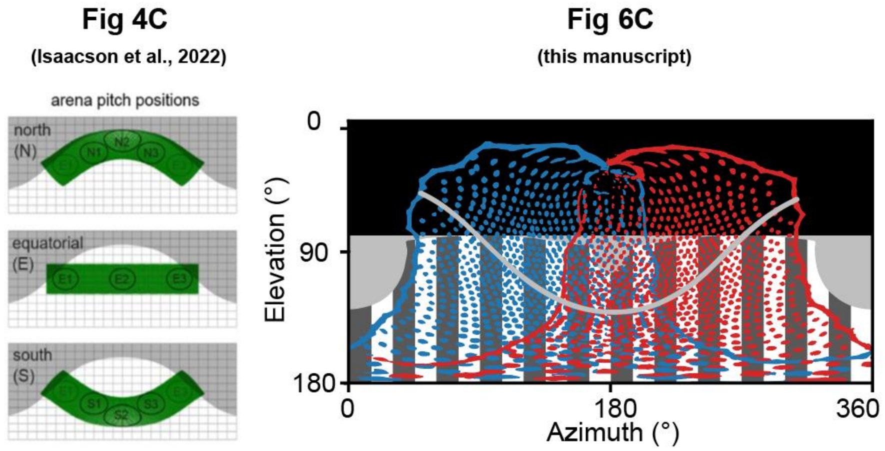

L257 change "velocity-depended" to "velocity-depending"

We reworded the sentence.

L441 add "to" in "comparable [to] those"

We added the missing word.
